# Gray-Scale Extraction of Bone Features from Chest Radiographs Based on Deep Learning Technique for Personal Identification and Classification in Forensic Medicine

**DOI:** 10.3390/diagnostics14161778

**Published:** 2024-08-15

**Authors:** Yeji Kim, Yongsu Yoon, Yusuke Matsunobu, Yosuke Usumoto, Nozomi Eto, Junji Morishita

**Affiliations:** 1 Department of Multidisciplinary Radiological Sciences, Graduate School of Dongseo University, 47 Jurye-ro, Sasang-gu, Busan 47011, Republic of Korea; 2 Department of Radiological Sciences, Fukuoka International University of Health and Welfare, 3-6-40, Momochihama, Sawara-ku, Fukuoka 814-0001, Japan; 3 Department of Forensic Pathology and Sciences, Graduate School of Medical Sciences, Kyushu University, 3-1-1, Maidashi, Higashi-ku, Fukuoka 812-8582, Japan

**Keywords:** bone extraction, personal identification, post-mortem computed tomography, deep learning, U-Net

## Abstract

Post-mortem (PM) imaging has potential for identifying individuals by comparing ante-mortem (AM) and PM images. Radiographic images of bones contain significant information for personal identification. However, PM images are affected by soft tissue decomposition; therefore, it is desirable to extract only images of bones that change little over time. This study evaluated the effectiveness of U-Net for bone image extraction from two-dimensional (2D) X-ray images. Two types of pseudo 2D X-ray images were created from the PM computed tomography (CT) volumetric data using ray-summation processing for training U-Net. One was a projection of all body tissues, and the other was a projection of only bones. The performance of the U-Net for bone extraction was evaluated using Intersection over Union, Dice coefficient, and the area under the receiver operating characteristic curve. Additionally, AM chest radiographs were used to evaluate its performance with real 2D images. Our results indicated that bones could be extracted visually and accurately from both AM and PM images using U-Net. The extracted bone images could provide useful information for personal identification in forensic pathology.

## 1. Introduction

Personal identification of an unknown individual is an important issue in forensic pathology [1]. Fingerprint recognition, DNA analysis, and dental information of the deceased are effective methods for gleaning sufficient evidence to identify unknown individuals or disaster victims. In addition, radiographic identification plays an important role in forensic science with the recent introduction of post-mortem computed tomography (PMCT). In contrast to autopsies, post-mortem (PM) radiographs are non-invasive and can be reinterpreted at any time. Since 1927, when Culbert and Law confirmed the identity of an unidentified person by matching ante-mortem (AM) and PM images, this technique has gradually gained popularity among forensic scientists. However, there are some significant differences between AM and PM images [2,3,4,5,6,7].

First, the properties of organs and soft tissues undergo considerable changes due to the effects of various chemical and physical processes that are initiated from the time of death. These bodily post-mortem changes include gravity-dependent changes (e.g., blood sedimentation, and livor mortis), decay of soft tissues, and rigor mortis. The decrease in lung volume and increase in intestinal gas caused by tissue decomposition due to post-mortem microbial activity result in major differences between AM and PM images, making it difficult to compare these images [8,9]. In 2007, Dedouit compared PMCT images and autopsy findings of cases in which only some bone tissue remained, owing to fire. He found that the two results were very similar and identified individuals using CT images [3]. Bone has been used to provide important information for identification of individuals, as its tissue characteristics change the least over time, and with environmental exposure. Therefore, to compare AM and PM images accurately, it is necessary to segment only the bone tissue signals.

Second, the medical equipment and scanning parameters used for imaging are different between AM and PM images. PMCT has become a standard procedure in many forensic institutes worldwide [10]. On the other hand, regarding AM clinical images, three-dimensional (3D) images are often not available, making it difficult to obtain both AMCT and PMCT images [11]. Chest radiography is a two-dimensional (2D) imaging technique, and it is the most commonly performed diagnostic X-ray examination. It is easier to collect these images than those obtained by CT or magnetic resonance imaging. Three-dimensional images can be converted to two-dimensional images through image processing, such as ray-summation projection. Pfaeffli et al. reported that 3D images can be converted to 2D for quick comparison [7]. Matsunobu et al. also compared PM and AM images of the same individual for personal identification under 3D and 2D conditions [11]. Their results revealed that the matching accuracy was comparable, but 3D processing was more time-consuming than 2D processing, and 2D matching was deemed to be more useful.

Bone segmentation techniques for 2D X-ray images include thresholding, edge-based, pattern-recovery-based, and atlas-based methods [12]. The thresholding technique, which is the most commonly employed method, requires an image with bimodal intensity distribution, and it is difficult to apply to 2D medical images. Moreover, the accuracy of segmentation depends on the distributions of pixel values and the user’s proficiency, because the threshold value is set by the operator [12,13].

Deep learning technologies have been introduced and widely adopted in medical image processing, with numerous studies confirming their success [14,15,16,17]. Deep learning employs neural networks with multiple layers to automatically learn and extract features from data, significantly enhancing performance across various tasks in medical imaging, including detection, classification, and segmentation. Detection identifies specific objects or anomalies within an image; classification assigns labels based on learned features; and segmentation aims to partition the image into distinct regions or structures, allowing for the separation of specific elements. In this context, deep learning provides a significant advantage for bone extraction. As mentioned earlier, traditional methods often struggle with accurately distinguishing bone structures from surrounding soft tissues due to their reliance on manual thresholds or predefined patterns. These techniques may fail to capture subtle variations in bone appearance or to deal effectively with noisy and complex backgrounds. Deep learning models, especially segmentation-focused networks like U-Net [18], overcome these limitations by learning from extensive datasets to automatically identify and isolate bone structures. This results in more precise and consistent bone segmentation, as deep learning algorithms can handle variability in image quality and anatomical differences better than traditional methods. For example, Wang et al. investigated how to mask the rib and clavicle regions of chest radiographs using multi-scale densely connected U-Net (MDU-Net) [15]. Ding et al. conducted segmentation of hand bones from X-ray images for bone age assessment using U-Net [16].

**Figure 1:**
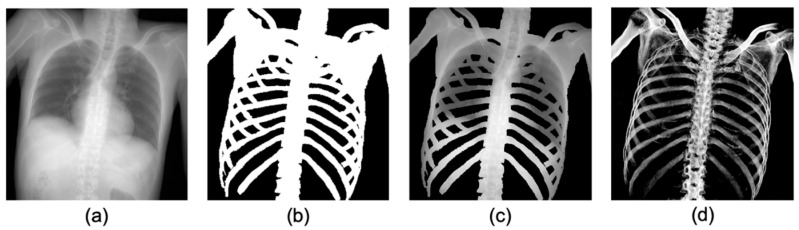
Comparison of datasets for bone extraction between diagnostic and forensic field. (**a**) An example of a radiograph-like image, (**b**) label image in diagnostic field: binary map of bone, (**c**) the corresponding parts for segmentation, including bone area according to label image in diagnostic field, and (**d**) gray-scaled bone image without soft tissues.

Although many studies have focused on bone segmentation in X-ray images, the majority of these studies have been aimed at diagnosis and treatments, such as computer-aided diagnosis. However, bone extraction for identification of the unknown deceased requires removal of soft tissue that shows the largest post-mortem changes. Training datasets for the deep learning model in the diagnostic and forensic field were compared in Figure 1. The binary maps (Figure 1b) of the bone on the X-ray image (Figure 1a) were used as a label image for training in the diagnosis field. An example of extracted regions corresponding to the label image is shown in Figure 1c [15]. This conventional method produces an output that contains the signals of soft tissues, such as the lungs and heart. However, as previously mentioned, unlike bone segmentation in the diagnostic field, for positive identification in the forensic field, it is necessary to create a gray-scaled bone-only image without overlap of soft tissue projections (Figure 1d) to allow comparison of AM and PM images under the same conditions. Therefore, the aim of this study is to develop a method to extract only the bone signals from pseudo 2D X-ray images projected from PMCT and AM clinical chest radiographs (CXR) using U-Net.

## 2. Materials and Methods

### 2.1. Overview of Proposed Method

**Figure 2:**
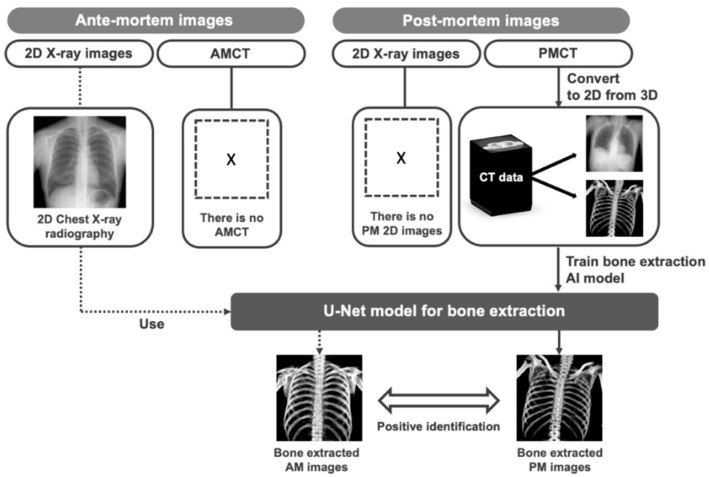
Overview of proposed method. Pseudo 2D X-ray images and corresponding bone images projected from CT volume data were used to train the deep learning model. Furthermore, clinical CXR images were also used to assess the extraction ability of the trained U-Net on real 2D X-ray images.

Our bone extraction method for positive identification in forensic pathology is summarized in Figure 2. To develop our proposed bone extraction method, 2D X-ray images and corresponding bone images were used as training materials for a deep learning model. Although bone images can be obtained through dual energy imaging, they may contain motion artifacts and organ information, which may result in inaccurate extraction components and affect the performance of the bone extraction model [19,20]. To address this issue, we utilized pseudo 2D X-ray images and corresponding bone images projected from PMCT volume data to train the deep learning model [19]. Additionally, to assess the extraction ability of the trained U-Net on real 2D X-ray images when AMCT data are not available for positive identification, we also utilized AM clinical CXR. It should be noted that this study specifically considered cases where PMCT images were available, and did not include cases where 2D images from PM were obtainable.

### 2.2. Image Database

The training data used in this study consisted of 110 PMCT images (men: 72; women: 38). The mean age at death was 52.1 ± 17.1 years (maximum: 89 years; minimum: 18 years; unknown: two cases). The cases with obvious body damage were not included in this study. All images were acquired using a 16-row multidetector CT scanner (ECLOS; Hitachi Medical Co., Tokyo, Japan) between 2014 and 2015. Scanning parameters were as follows: tube voltage 120 kV, tube current 250 mAs, slice thickness 1.0 mm, and pixel size 0.98 mm [11,21]. Data splitting was performed for U-Net training. We used 100 cases for training and 10 cases for testing. Furthermore, 20% of the training data was used for validation during model training.

Additionally, to address the limitation of the testing dataset, clinical CXRs used to assess the extraction ability on real 2D X-ray images were obtained using a CR system (FUJIFILM Corporation, Tokyo, Japan) as part of a mass survey of lung cancers in Iwate Prefecture, Japan [21,22]. The age range of the patient at the time of acquisition was from the 30 s to the 70 s. The images had a matrix size of 1760 × 1760 (0.2 mm pixel size), and were resized to 256 × 256 by bilinear interpolation.

The study was approved by the Institutional Review Board at Kyushu University, Japan. The PMCT component received approval number #2017-27-285, and the clinical CXRs component received approval number #2020-235.

### 2.3. Pre-Processing: Producing Training Datasets from PMCT Images

**Figure 3:**
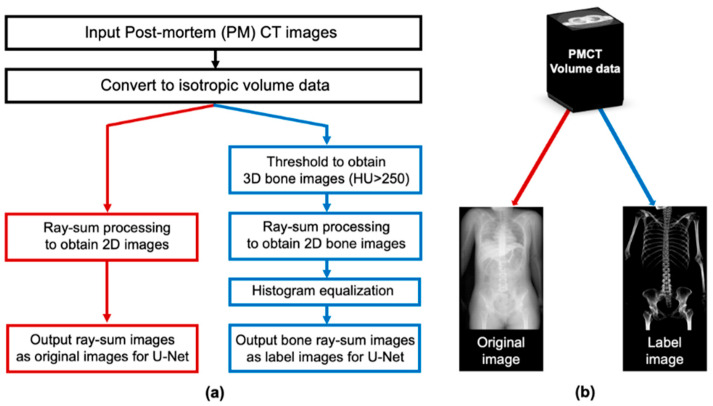
Process for obtaining training datasets from the PMCT images. (**a**) Flowchart. (**b**) The input image, a pseudo 2D X-ray image, was generated according to the red line, while the corresponding bone image was generated following the blue line.

A flowchart for producing pseudo 2D X-ray images and corresponding bone images is illustrated in Figure 3. First, the slice from the shoulder to the upper femur was converted into isotropic volume data for each case. The CT values of each voxel were then summed along a projection line in the anterior–posterior projection. Two types of radiographic images were created for each case. One was a pseudo 2D X-ray image that projected all signals in the body, including bone and soft tissues, and the other was a bone image that projected only bone by thresholding at a CT value (250 Hounsfield Unit [HU]). This threshold was empirically determined to extract only bone structures [11]. In addition, histogram equalization was performed on the bone image to improve contrast. These images, with a size of approximately 512 × 800 pixels, were used as input and label images for deep learning training.

To expand the dataset, image cropping was used as an offline data augmentation technique. The 2D pseudo images were cropped to 256 × 256 pixels, moving 50 pixels from the upper left to the lower right. This offline process created all augmented images before training. Based on empirical analysis, it was determined that approximately 6000 sets of data are necessary for effective training of the model. The training and validation datasets consisted of 4668 and 1152 sets, respectively. Another 582 sets were used as the test dataset, as shown in Table 1. The cases used for training, validation, and testing were not mixed. Moreover, the pixel values of the input images and label images were normalized to bring them to the 0–1 range.

### 2.4. U-Net Architecture

**Figure 4:**
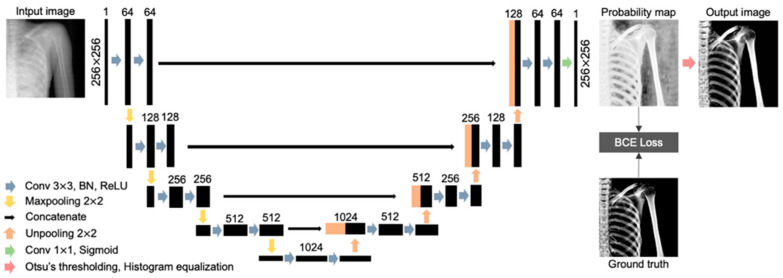
Overall architecture of the proposed bone extraction model of U-Net.

In this study, U-Net was employed for gray-scale extraction of bone features. U-Net is one of the fully convolution network models proposed for the purpose of image segmentation, and has been widely utilized in segmentation research because its relatively simple architecture makes it easy to implement and reproduce, confirming its usefulness. U-Net comprises a contracting path and an expanding path, and is named after the letter “U” in the alphabet, as shown in Figure 4. In the contracting path, down-sampling is repeated to generate feature maps. Each down-sampling operation consists of a 3 × 3 convolution layer with a rectified linear unit (ReLU) and batch normalization (BN), and a 2 × 2 Max-pooling layer. Repeated down-sampling reduces the size of the feature map and doubles the number of channels. In the expanding path, up-sampling is performed by repeating the 3 × 3 convolution layer and the 2 × 2 un-pooling layer to generate the resulting image of the region extraction from the feature map generated in the contracting path. By connecting the feature map of the contracting path to the expanding path with a skip connection, global and local features can be controlled [18,23]. Finally, the output layer consisting of 1 × 1 convolution generates a probability map where the output of each pixel is a value between 0 and 1, by applying the sigmoid function [24].

Transfer learning was not utilized in this study because our focus was on gray-scale extraction of bone, whereas binary segmentation often benefits more from pre-trained models for similar tasks. The model was trained for 30 epochs using the Adam optimizer with a learning rate of 0.001 (Table 2).

As the loss function of the network, we used and minimized the binary cross-entropy function. The calculation formula for binary cross-entropy is shown as follows:

(1) $$BCE\;Loss\left(\hat{y},y\right)=-\left(y\times \log\hat{y}+\left(1-y\right)\times \log\left(1-\hat{y}\right)\right),$$

y represents the pixel of each pixel in the label image converted to a value between 0 and 1, and $\hat{y}$ represents the output from the continuous sigmoid function between 0 and 1. In deep learning, the parameters are adjusted so that the loss function becomes as small as possible.

In the probability map, bone is output as a value close to 1, and non-bone regions as a value close to 0. In U-Net, a fixed threshold, such as 0.5, is commonly used to convert the probability map into a binary map of black and white. However, in this study, the optimal threshold for each output image was automatically determined by applying Otsu’s algorithm, which separates the image into the following two classes: background and bone [25,26]. The non-bone region signal was eliminated by replacing pixel values below the threshold determined by Otsu’s algorithm with 0. Additionally, pixel values above the threshold (bone region) were converted to values between 0 and 255 by histogram equalization.

The proposed method was developed and evaluated using Python 3.9.7 on a workstation (central processing unit: Intel Core i7-8750H processor; memory: 16 GB; graphics processing unit: NVIDIA GeForce GTX 1060).

### 2.5. Evaluation of Bone Extraction Performance by U-Net

Both the ground truth and the predicted images generated using U-Net are grayscale images with continuous pixel values. Therefore, in this study, we conducted two types of evaluations, as follows: one based on the segmentation of bone regions considering only the presence or absence of bones, and another based on the evaluation of the intensity distribution of bone signals considering the continuous pixel value distribution of bones.

#### 2.5.1. Evaluation on the Extracted Bone Regions

The segmentation performance of the bone region was evaluated using Intersection over Union (IoU), Dice coefficient, sensitivity, and specificity. The ground truth and predicted image were composed of pixel values ranging from 0 to 255. Since the background region of the ground truth image contains random noise with pixel values less than 40, the criterion for bone region determination was set at pixel values of 40 or higher. IoU and Dice coefficient are calculated based on the number of overlapping pixels between the ground truth and predicted image [27]. IoU and Dice coefficient were calculated using the following equation:

(2) $$IoU=\frac{TP}{TP+FP+FN}$$

(3) $$Dice\;coefficient=\frac{2\times TP}{2\times TP+FP+FN}$$

TP (true-positive) represents the number of pixels where the bone regions of the ground truth and the predicted image overlap. FP (false-positive) represents the number of pixels where the bone regions are extracted only from the predicted image. FN (false-negative) represents the number of pixels where the bone regions are extracted only from the ground truth image. The formulas for sensitivity and specificity are shown as follows:

(4) $$Sensitivity=\frac{TP}{TP+FN}$$

(5) $$Specificity=\frac{TN}{TN+FP}$$

TN (true-negative) represents the number of pixels in the region where no bones are extracted in both the ground truth image and the predicted image [16].

#### 2.5.2. Evaluation of Bone Signal Intensity Distribution

To evaluate whether the distribution of bone signal intensity is correctly predicted, we calculated the sensitivity and specificity for all thresholds by changing the threshold criteria from −1 to 256, which was set to a pixel value of 40 in Section 2.5.1. Since both the ground truth and predicted images have pixel values ranging from 0 to 255, setting the threshold to −1 would result in the entire image being classified as TP, while setting it to 256 would classify the entire image as TN. Based on the sensitivity and specificity calculated for each threshold, a receiver operating characteristic (ROC) curve was written. The vertical axis of the ROC curve is the true-positive fraction (TPF: sensitivity), and the horizontal axis is the false-positive fraction (FPF: 1 − specificity). As a quantitative evaluation, we also calculated the area under the ROC curve (AUC) [28,29].

## 3. Results

**Figure 5:**
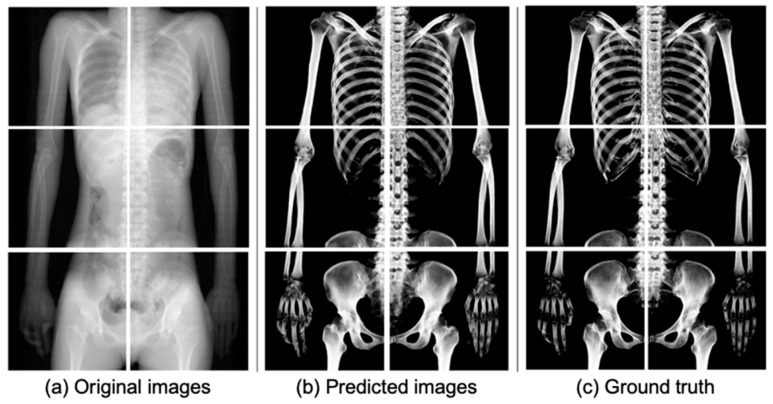
Comparison of input, predicted, and ground truth images. Pseudo 2D X-ray images used as (**a**) input images were generated by ray-summation projecting all signals from the 3D volume data, including both bone and soft tissues. (**b**) Predicted bone images were obtained using the trained U-Net model. (**c**) Ground truth images were generated by ray-summation projecting only the bone components from the original PMCT data, with bone regions identified using a thresholding technique based on CT values.

Figure 5 shows the results of extracting bones from the input images using the trained U-Net in this study. By comparing the predicted images (Figure 5b) with the ground truths (Figure 5c), the bone could be extracted quite accurately in the predicted images.

### 3.1. Results of Bone Region Extraction Evaluation

The evaluation results of bone region segmentation, considering only the presence or absence of bone as described in Section 2.5.1, are shown in Table 3. The means of the IoU, Dice coefficient, sensitivity, and specificity were 0.834, 0.909, 0.944, and 0.895, respectively, when the threshold was set to 40.

### 3.2. Results of Bone Signal Intensity Distribution Analysis

**Figure 6:**
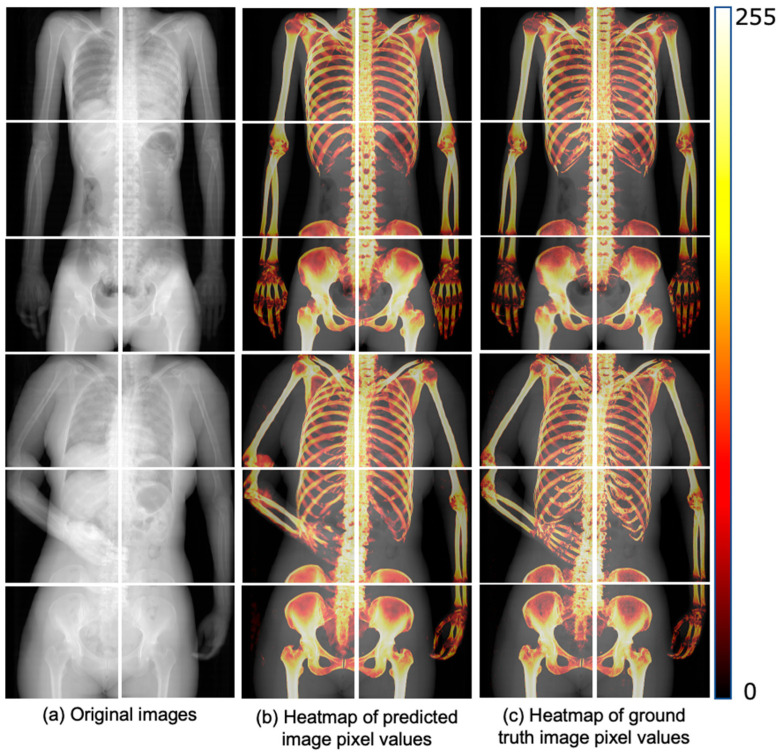
Comparison of pixel value distribution between predicted and ground-truth images. (**a**) Shows the original pseudo 2D X-ray images used as input. (**b**) Displays the heatmap of pixel values from the bone images predicted by the trained U-Net model, while (**c**) shows the heatmap of pixel values from the ground truth images. The heatmaps in (**b**,**c**) are used to visually compare the accuracy of the model’s predictions.

**Figure 7:**
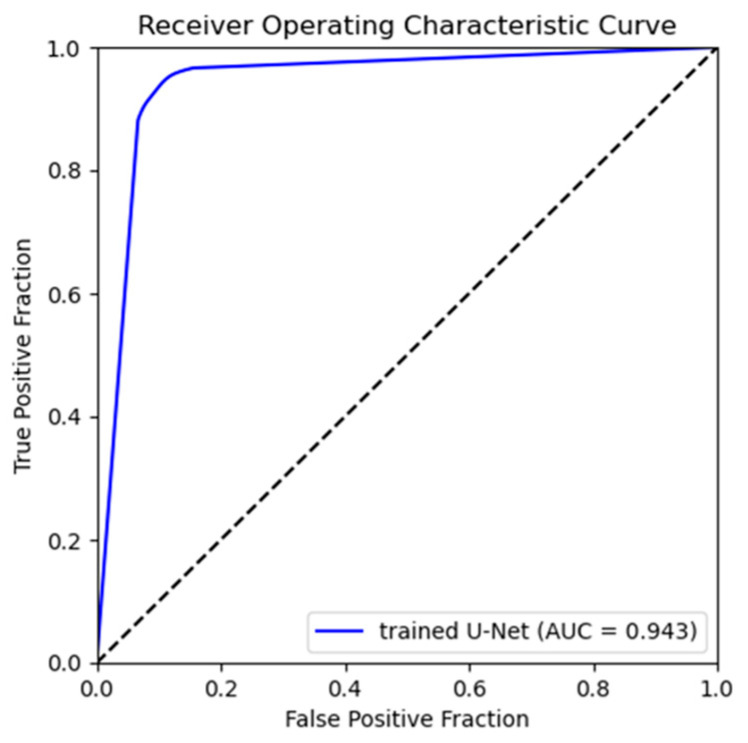
The ROC curve to assess the reproducibility of the signal intensity distribution for bone extraction. TPF and FPF were calculated for all thresholds.

Figure 6 shows a heatmap visualization of the predicted and ground truth image pixel values colored in the range of 0 to 255 for the trained U-Net. The color change due to the pixel value distribution is shown on the right-side bar of Figure 6. The pixel value distributions of the predicted and ground truth images are similar. Additionally, Figure 7 presents the ROC curve created from the true-positive fraction (TPF) and false-positive fraction (FPF), calculated as described in Section 2.5.2. The AUC value, which represents the area under the ROC curve, was high, at 0.943.

### 3.3. The Performance of Bone Extraction on Real 2D X-ray Images (CXRs)

**Figure 8:**
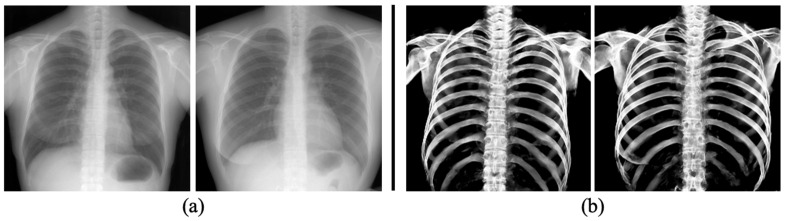
Samples of bone extraction results (**b**) of trained U-Net from clinical CXRs (**a**).

To evaluate the capability of the proposed model to extract bones from real 2D X-ray images, we performed tests using clinical CXRs. The clinical CXRs were used as input images, and their corresponding bone extraction images are depicted in Figure 8. Bone signals were clearly extracted, even though the images were acquired from a different modality than the data used for model training.

## 4. Discussion

This study attempted bone extraction from 2D X-ray images by using the U-Net algorithm, and evaluated the effectiveness of this method. We found promising results, with good visual performance and reasonably high index measures.

The evaluation metrics, including IoU, Dice coefficient, sensitivity, and specificity, were employed to assess the accuracy of the extracted bone region. The average values obtained were 0.834, 0.909, 0.944, and 0.895, respectively (Table 3). These results exceeded the values reported in the previous study by Wang et al. [15], where the extraction of chest bones was performed using U-Net. It is especially the case that the proposed model achieved excellent performance in extracting rib bones, despite their thin structures and low contrast due to organ overlap, such as the heart. Furthermore, the predicted images generated by U-Net (Figure 5b and Figure 8b) specifically captured bone features while excluding rib cartilage signals. Conversely, the ground truth images (Figure 5c) created using a CT value thresholding failed to separate calcified rib cartilage, which exhibits higher CT values, from the bones. As rib cartilage is primarily composed of cartilaginous tissue, and is subject to significant PM changes, it has limited utility for personal identification in the field of forensic pathology. Our proposed method enables the creation and comparison of bone images from 2D X-ray images, even in scenarios where acquiring both AM and PM 3D images is challenging. 

In this study, we utilized gray-scaled label images instead of binary images, allowing us to capture the signal intensities of each bone tissue. The difference between the output of U-Net for each pixel and the corresponding label was calculated using the binary cross-entropy loss function. This loss function guided the training process, minimizing the discrepancy between the output and label image. Consequently, the AUC was calculated as an indicator of how closely the intensity distribution of bone tissue in the predicted image resembles the ground truth image, and it yielded a high value of 0.943. This result suggests that the model accurately extracted the intensity distribution of bone signals. The intensity distribution of bone signals contains essential information for assessing factors such as bone density and the formation of bone spurs, which can potentially be utilized for estimating the age of unidentified remains [30,31]. Furthermore, gray-scaled bone images provide a versatile approach that can be employed, even in situations where both AM and PMCT images are unavailable. This approach can contribute to the development of more generalized and novel methods for personal identification.

**Figure 9:**
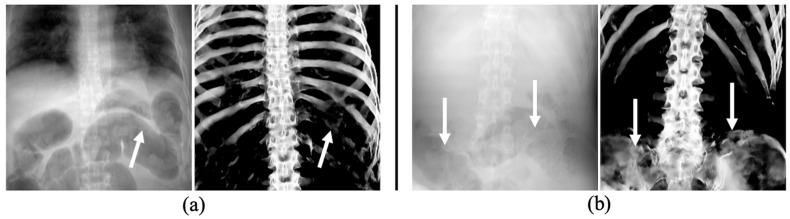
Examples with artifacts in the ribs (**a**) and pelvic region (**b**) due to large amounts of intestinal gas, as indicated by white arrows.

In the abdominal regions of two cases shown in Figure 9, distortions were observed in the predicted images of rib and pelvic regions, resulting in evaluation metric values approximately 0.2 lower than average values (Table 3). These distortions were mainly attributed to the presence of overlapping gas within the intestines. The high contrast between the gas shadows and the adjacent soft tissue boundaries is believed to be the underlying cause of these distortions. Such subtle differences in images can potentially lead to misidentification in personal identification. In a previous study, Shimizu et al. confirmed that using the upper chest as a biological fingerprint, which is less affected by the breathing phase, in patient recognition could increase the accuracy of identification compared to using the entire chest region [32]. Based on this research, it is necessary to examine whether AM and PM images can be matched using only the upper chest regions, which are less affected by intestinal gas. Future research should be conducted in parallel to improve the extraction performance in areas where the intestinal gas and ribs overlap.

## 5. Limitations and Future Directions

It is important to acknowledge the limitations of this study. Firstly, the number of cases tested was small. Additionally, including numerous unknown images could yield different findings. Secondly, we did not compare the extraction performance of our deep learning models to other models designed for segmentation purposes because our study aimed to verify the potential for gray-scale extraction of bone features from 2D medical images, rather than for personal identification and classification, at this time. Therefore, personal identification or classification using the proposed method in this study should be pursued in future research.

Some anatomical characteristics, referred to as “Biological Fingerprints (BFs)”, on chest radiographs—such as the lung apex, superior mediastinum, right lower lung, whole lung field, and cardiac shadow—were utilized for positive identification in the Radiology Information System to prevent misfiling errors [5,6,22,32,33,34,35]. Previous studies have shown that BFs are effective for personal identification and reducing misfiling errors in Picture Archiving and Communication Systems (PACS). However, most BFs are found in soft tissue or high-contrast regions such as the lungs, which are susceptible to post-mortem changes. Therefore, we will apply the proposed bone extraction method to chest radiographs and assess its utility for personal identification and classification in future studies. 

Additionally, we aim to explore bone-originated anatomical landmarks, such as the ribs and clavicles, which are key points for personal identification. The proposed model has potential applications in mass casualty events, where it can support the rapid identification of numerous victims. Moreover, it may enhance the identification process for decomposed or damaged remains, which are challenging for conventional methods. Therefore, this type of research can establish a standard protocol to facilitate high-speed identification, particularly for forensic pathology and radiographic identification.

## 6. Conclusions

Our proposed method successfully extracted bone signals from pseudo 2D X-ray images projected from PMCT data and AM clinical CXR using a U-Net-based deep learning model. By employing the trained U-Net for bone extraction, it was possible to obtain gray-scaled bone images from 2D X-ray images where bone extraction is challenging compared to 3D images. The proposed method could facilitate obtaining extracted bone-only images to allow for comparison to AM images, and to facilitate personal identification and classification.

## Figures and Tables

**Table 1:** Number of images before and after data augmentation.

	Before Augmentation	After Augmentation
Training	80	4668
Validation	20	1152
Test	10	582
Total	110	6402

**Table 2:** List of hyperparameter values used for U-Net.

Hyperparameter	Value
Epochs	30
Batch size	16
Learning rate	0.001
Activation	Sigmoid
Optimizer	Adam
Loss function	Binary cross-entropy

**Table 3:** Evaluation results on the extracted bone regions.

	IoU	Dice	Sensitivity	Specificity
Average	0.834	0.909	0.944	0.895
Maximum	0.920	0.959	0.997	0.986
Minimum	0.638	0.779	0.857	0.688

## Data Availability

All data generated or analyzed during this study are included in this published article or available from the corresponding author on reasonable request.
